# Modification of Diet to Reduce the Stemness and Tumorigenicity of Murine and Human Intestinal Cells

**DOI:** 10.1002/mnfr.202200234

**Published:** 2022-08-31

**Authors:** Stephanie May, Kirsty R. Greenow, Adam T. Higgins, Anna V. Derrick, Elaine Taylor, Pan Pan, Maria Konstantinou, Colin Nixon, Thomas E. Wooley, Owen J. Sansom, Li‐Shu Wang, Lee Parry

**Affiliations:** ^1^ European Cancer Stem Cell Research Institute School of Biosciences Cardiff University Cardiff CF24 4HQ UK; ^2^ Division of Haematology and Oncology Medical College of Wisconsin 8701 Watertown Plank Rd Milwaukee WI 53226 USA; ^3^ CRUK Beatson Institute Switchback Road, Bearsden Glasgow G61 1BD UK; ^4^ School of Mathematics Cardiff University Senghennydd Road Cardiff CF24 4AG UK; ^5^ Insitute of Cancer Sciences University of Glasgow Switchback Road, Bearsden Glasgow G61 1QH UK; ^6^ Wales Cancer Research Centre University Hospital of Wales Room 1TB2 31, First Floor Main Building Cardiff CF14 4XN UK

**Keywords:** anthocyanins, black raspberries, chemoprevention, colorectal cancer, intestinal stem cells

## Abstract

**Scope:**

Black raspberries (BRBs) have colorectal cancer (CRC) chemo‐preventative effects. As CRC originates from an intestinal stem cell (ISC) this study has investigated the impact of BRBs on normal and mutant ISCs.

**Methods and results:**

Mice with an inducible *Apc^fl^* mutation in either the ISC (*Lgr5CreER^T2^*) or intestinal crypt (*AhCre/VillinCreER^T2^*) are fed a control or 10% BRB‐supplemented diet. This study uses immunohistochemistry, gene expression analysis, and organoid culture to evaluate the effect of BRBs on intestinal homeostasis. RNAscope is performed for ISC markers on CRC adjacent normal colonic tissue pre and post BRB intervention from patients. 10% BRB diet has no overt effect on murine intestinal homeostasis, despite a reduced stem cell number. Following *Apc* ISC deletion, BRB diet extends lifespan and reduces tumor area. In the *AhCre* model, BRB diet attenuates the “crypt‐progenitor” phenotype and reduces ISC marker gene expression. In ex vivo culture BRBs reduce the self‐renewal capacity of murine and human *Apc* deficient organoids. Finally, the study observes a reduction in ISC marker gene expression in adjacent normal crypts following introduction of BRBs to the human bowel.

**Conclusion:**

BRBs play a role in CRC chemoprevention by protectively regulating the ISC compartment and further supports the use of BRBs in CRC prevention.

## Graphical abstract

Colorectal cancer (CRC) develops from a mutated intestinal stem cell (ISC). Using CRC/ISC mouse models and patients we demonstrate that black raspberries (BRBs) reduces ISC number and gene expression, inhibits human CRC organoid self‐renewal and extends CRC/ISC mouse survival. Suggesting the link between BRBs and cancer prevention maybe due, at least, in part to an impact on the ISCs. ≈

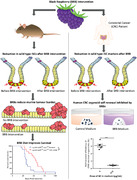

## 1 Introduction

CRCis linked to dietary choices and is the 2nd leading cause of malignancy‐related deaths in the Western world.^[^ ^1^ ^]^ With ≈50% of cases thought to be preventable by lifestyle choices^[^ ^2^ ^]^ there is a need to understand how diet impacts upon the normal intestine. Ultimately the interaction of an individual's diet with their intestine impacts on theirISCs which maintain the epithelial barrier and are considered the “cell of origin” of CRC.^[^ ^3^ ^]^ Current research examining a high‐fat diet (HFD) and CRC has demonstrated it increases ISC numbers and the risk of oncogenic transformation;^[^ ^4^ ^]^ setting a precedent for the ISC impact of diet. Potentially dietary components associated with reduced CRC risk may be linked to a reduction in ISC activity in the healthy intestine. Previous studies have demonstrated that administration of BRBs inhibited tumorigenesis in: *Apc*1638^+/−^ mice, inflammation‐driven CRC in *Muc2* ^−/−^ mice,^[^ ^5^ ^]^ CRC patients,^[^ ^6^ ^]^ and lead to polyp regression in familial adenomatous polyposis (FAP) patients.^[^ ^7^ ^]^ FAP patients inherit a mutated copy of the *APC* gene, *APC* loss in an ISC activates the WNT pathway, and is the earliest known event in CRC; a key feature of inherited and sporadic (≈90%) CRCs. In this study we used pre‐clinical ex vivo and in vivo models and material from a CRC BRB clinical intervention trial to establish whether BRBs impact on CRC via modulation of the ISC pool.

## 2 Results

### 2.1 Dietary BRBs Extend Survival of *Lgr5CreER^T2^ Apc^fl/fl^* Mice

**Figure 1:**
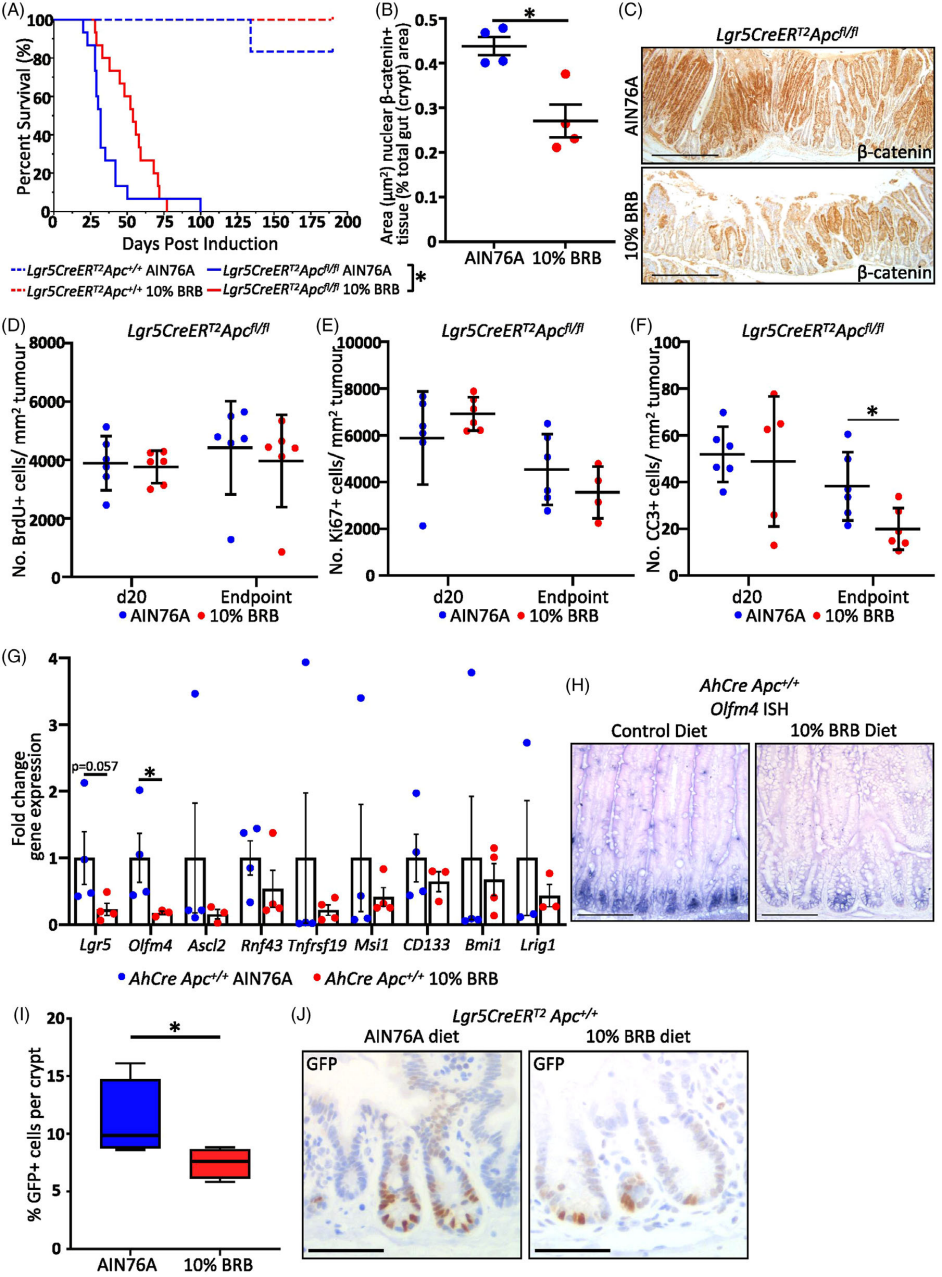
BRB diet suppresses intestinal tumorigenesis and ISCs in the *Lgr5CreER^T2^* and *AhCre Apc^fl/fl^* mouse. A) 10% BRB diet significantly extends survival following ISC *Apc* deletion. B and C) 20 d.p.i BRB diet significantly reduced nuclear β‐catenin+ (brown) lesions in the intestine (500 µm). BRB diet does not affect tumor cell proliferation at 20 d.p.i or at endpoint D and E) but cleaved caspase 3 (CC3) cell death is significantly reduced in endpoint tumors F). G) BRB diet suppresses ISC gene expression in *AhCreApc^+/+^* mice (mean ± SEM). H) Representative in situ images demonstrating BRB induced *Olfm4* reduction in WT intestinal crypts (100 µm). I and J) BRB diet reduces the number of GFP^+^ ISCs (brown) in WT crypts of mice (50 µm).

To establish whether BRBs could impact on ISCs, cohorts of *Lgr5CreER^T2^Apc^+/+^* and *Lgr5CreER^T2^Apc^fl/fl^* mice were randomly assigned to either control or 10% BRB diet from 2 weeks prior to Cre induced ISC *Apc* deletion (Figure S1A, Supporting Information). Analysis of weight change demonstrated no effect of BRB administration in either cohort (Figure S1B,C). At 190 days post induction (d.p.i.) there was no significant difference in the survival of the *Lgr5CreER^T2^Apc^+/+^* cohorts (**Figure** **1**A). In contrast, the BRB diet significantly increased survival of *Lgr5CreER^T2^Apc^fl/fl^* mice (Figure 1A). With the area of nuclear β‐catenin positive lesions (a surrogate marker for *Apc* loss) at 20 d.p.i (*N* = 4) significantly reduced in the BRB cohort (Figure 1B,C). The reduction in lesions at 20 d.p.i in BRB treated mice was not the result of reduced proliferation or increased cell death as the number of BrdU+, Ki67+ (proliferation), and CC3+ (Cleaved Caspase‐3, cell death) cells were unaltered between the two cohorts (Figure 1D–F). However, in endpoint tumors, there was no change in proliferation but a significant reduction in CC3+ cell death (Figure 1D–F). To assess the impact of BRBs on the ISC population we used the *AhCreApc^fl/fl^* ^[^ ^8^ ^]^ acute model of *Apc* loss to amplify alterations to the ISCs and Wnt signaling pathway. Following induction, *AhCre* driven *Apc* loss acutely activates the canonical Wnt pathway in all cells of the intestinal crypt (except Paneth cells). Mice were placed on diets 2‐weeks prior to *Apc* loss and harvested 5 d.p.i. In *Apc^+/+^* mice gene expression analysis of a range of putative ISC markers indicated BRBs significantly reduced expression of the key ISC marker *Olfm4* and a non‐significant >4 fold reduction in *Lgr5* (Figure 1G,H). A similar pattern was observed in the *AhCreApc^fl/fl^* BRB treated mice with significant reduction in *Olfm4*, a decrease in *Lgr5* expression and significant upregulation of *Ascl2* (Figure S1D,E). The absence of significant upregulation of the Wnt target *Lgr5*, in this model of acute Wnt activation is consistent with an ISC role for BRBs and further supported by the increase in the Wnt target *Ascl2*. *Ascl2* plays a role in the dedifferentiation of neighboring non‐ISC epithelial cells to an ISC state; a mechanism for replenishing a depleted *Lgr5* stem cell pool.^[^ ^9^ ^]^ As a HFD promotes tumorigenesis in mice by increasing ISC number^[^ ^4^ ^]^ we next determined whether BRBs reduced the number of normal ISCs. Using the *Lgr5CreER^T2^Apc^+/+^* mouse we demonstrated that a 2‐week BRB diet significantly reduces the ratio of ISCs:non‐ISCs in the normal crypt (Figure 1I,J). Suggesting that in this model BRBs may prevent intestinal tumorigenesis by decreasing the number of *Lgr5+* ISCs prior to and following *Apc* loss.

### 2.2 BRB Exposure Modifies the *Apc* Deficient *“*crypt‐progenitor phenotype*”*

**Figure 2:**
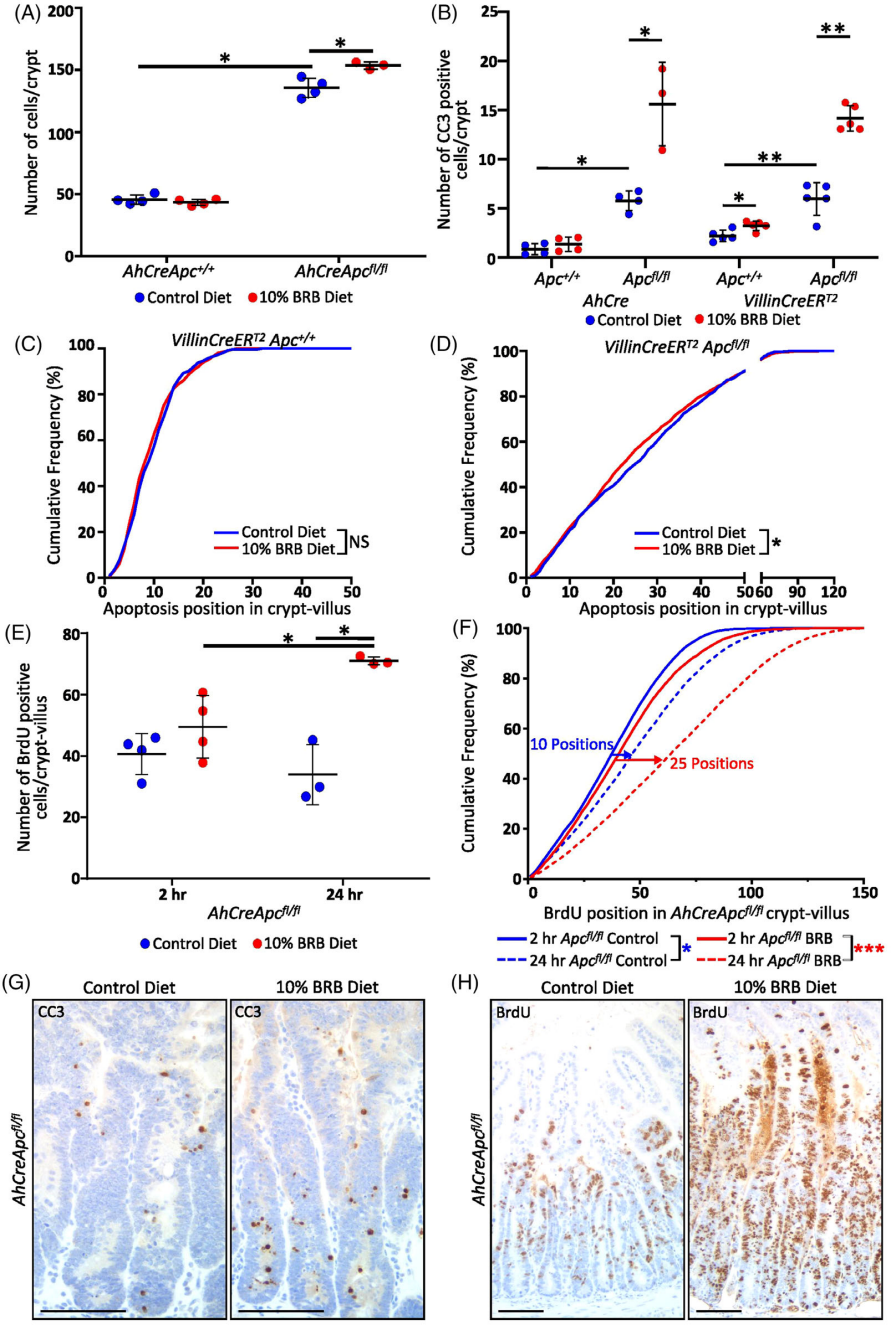
BRB diet alters the *Apc* deficient intestinal “crypt‐progenitor phenotype.” In the *Apc* deficient crypt BRB diet increases the number of A) cells and B) cleaved Caspase‐3 (CC3) apoptotic cells (in *AhCre* and *VillinCreER^T2^ Apc^fl/fl^* mice) (*N* = 3–5 mice). BRB diet did not affect the amount or position in which apoptosis occurs in *Apc^+/+^* crypts (B–C; NS = not significant) but did induce more apoptosis between cell positions 15 and 40 along the crypt‐villus axis in *VillinCreER^T2^ Apc^fl/fl^* mice compared to control treated mice D) (*N* = 5 mice). E) BRB diet also increases the number of proliferating cells in *AhCreApc^fl/fl^* mice; with no effect on the *AhCreApc^+/+^* intestine crypts (*N* = 3–5 mice). F) Cumulative frequency graph indicating that BRB diet stimulates migration of BrdU+ cells in the *Apc* deficient crypt (*N* = 3–4 mice). Representative images of *AhCreApc^fl/fl^* small intestine showing an increase in CC3 apoptotic cells (G; CC3‐brown; 100 µm) and proliferating cells with altered distribution 24 h following BrdU labeling (H; brown; 100 µm).

**Figure 3:**
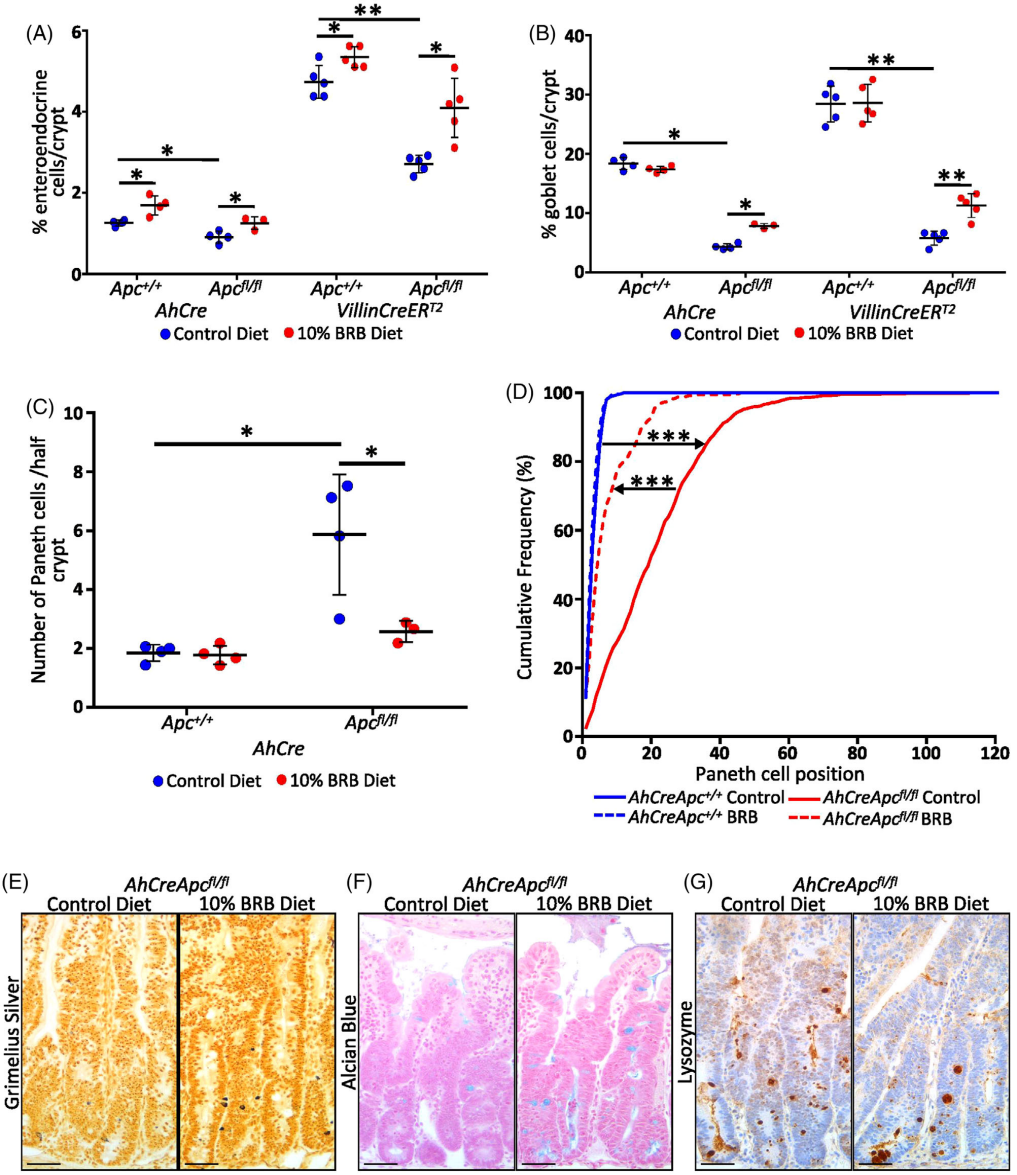
BRB diet restores differentiation in the induced *AhCre* and *VillinCreER^T2^ Apc^fl/fl^* intestinal crypt. In the *Apc^fl/fl^* crypt BRB diet increases the numbers of A) enteroendocrine and B) goblet cells (and *Apc^+/+^*) and partially restores the number C) and position D) of Paneth cells in *AhCreApc^fl/fl^* crypts (*N* = 3–5 mice). Representative images of enteroendocrine cells (E; black), goblet cells (F; blue), and Paneth cells (G; brown) in induced *AhCreApc^fl/fl^* crypts following BRB (50 µm).

As BRBs impacted the ISC population we assessed the effect of BRBs on *Apc^−/−^* crypt cell dynamics with the *AhCreApc^fl/fl^* model. Despite a reduction in the number of ISCs, BRB exposure had no significant effect on crypt cell number, mitosis, proliferation or cell death in WT mice, except for an increase in CC3+ cells in *VillinCreER^T2^Apc^+/+^* crypts (**Figure** **2**A–C). To determine whether the BRB diet specifically targeted the ISCs for cell death we quantified the position of apoptotic CC3+ cells in the *VillinCreERT2Apc^+/+^* mice. The position of CC3+ cells were not different between control and BRB diet, and the majority of cell death occurred above cell position 5, suggesting that cell death mostly occurred outside the stem cell population (Figure 2C). The “crypt‐progenitor phenotype” is a term used to characterize the immediate consequences of *Apc* loss within the intestinal crypt. Following the loss of *Apc*, a rapid localization of β‐catenin to the nucleus results in an Wnt‐activated, elongated, hyper‐proliferative crypt due to an increase in ISC and Paneth cells (and mislocalization of these cell types) concomitantly with increased cell death, failed differentiation, and aberrant migration.^[^ ^10^ ^]^ In contrast to the *Apc^+/+^* setting, BRB diet significantly modified the *Apc^−/−^* “crypt‐progenitor phenotype” (Figure 2A,B,D–H).^[^ ^10^ ^]^ With a BRB diet further elevating the increase in total number of cells/crypts, proliferating cells and apoptotic bodies and restoring migration along the crypt‐villus axis (Figure 2A,B,D–H). In the *Apc^fl/fl^* crypts, whereby loss of *Apc* has been shown to increase the number and size of the stem cell population^[^ ^10^ ^]^ the position of cell death was slightly altered in BRB fed mice, such that apoptosis was shifted by approximately three cell positions lower (towards the crypt base) than that in the control fed mice, however, cell death in both dietary settings occurred equally within the first 15 cell positions (Figure 2D), suggesting that BRBs do not selectively target crypt base columnar stem cells for cell death. Due to the increase in stem‐like cells in *Apc^fl/fl^* crypts, it is plausible that BRBs push these stem‐like cells to apoptosis‐mediated cell death and this could aid in the attenuation of the “crypt‐progenitor phenotype.” The restoration of cell migration indicated that BRBs were influencing the signaling pathways that direct differentiation, both of which are inhibited in the *AhCreApc^fl/fl^* model. In WT mice, BRBs significantly increased the nutrient sensing enteroendocrine cells with no effect on goblet or Paneth cell numbers (**Figure** **3**A–C). With BRB diet attenuating the *Apc^fl/fl^* phenotype,^[^ ^10^ ^]^ significantly increasing enteroendocrine (Figure 3A,E) and goblet cells (Figure 3B,F), while Paneth cells decreased in number (Figure 3C,G) and localize back towards the base of the crypt (Figure 3D). This increase in the number of apoptotic CC3, enteroendocrine, and goblet cells was confirmed using the *VillinCreER^T2^Apc^fl/fl^* model, which deletes *Apc* in the entire crypt‐villus compartment, including the Wnt3A producing Paneth cells (Figures 2B and 3A,B). As BRBs increased apoptosis in *AhCre*, *LgrCreER^T2^*, and *VillinCreER^T2^ Apc^fl/fl^* mice we next sought to establish whether the increase in survival is due to increased *Apc^−/−^* cell death rather than a reduction in ISCs prior to *Apc* loss. To establish this, we used the following assumptions: 1) each *Apc^−/−^* cell has a production rate of *Pr* (BrdU+ cells/total cells) and an apoptotic rate *Ap* (CC3+ cells/total cells), and 2) these rates are the same over all cells and constant over the time of the experiment; thus the difference between *Pr* and *Ap* indicates the net growth rate of the cells. For control diet *Pr* = 0.25 (33.94)/(135.7) and *Ap* = 0.04 (5.78/135.7) thus *Pr‐Ap* = 0.21; for BRB diet, *Pr* = 0.46 (71.05/153.7) and *Ap* = 0.1 (15.61/153.7) thus Pr‐Ap = 0.36. Hence, the net growth rate of the *Apc^−/−^* population has increased from 0.21 to 0.36 on a BRB diet; in contrast to the observed reduction in area of β‐catenin positive lesions (Figure 1B,C). Together this supports the rationale that in *Lgr5CreER^T2^Apc^fl/fl^* mice a BRB diet reduces the number of ISCs required to maintain homeostasis, thus limiting the number of *Apc^−/−^* ISCs following induction which alongside BRB suppression of the *Apc^fl/fl^* “crypt‐progenitor phenotype” manifests as reduced tumorigenesis and increased lifespan.

### 2.3 BRB‐Derived Anthocyanins Reduce Self‐Renewal Efficiency of *Apc* Deficient Cells

**Figure 4:**
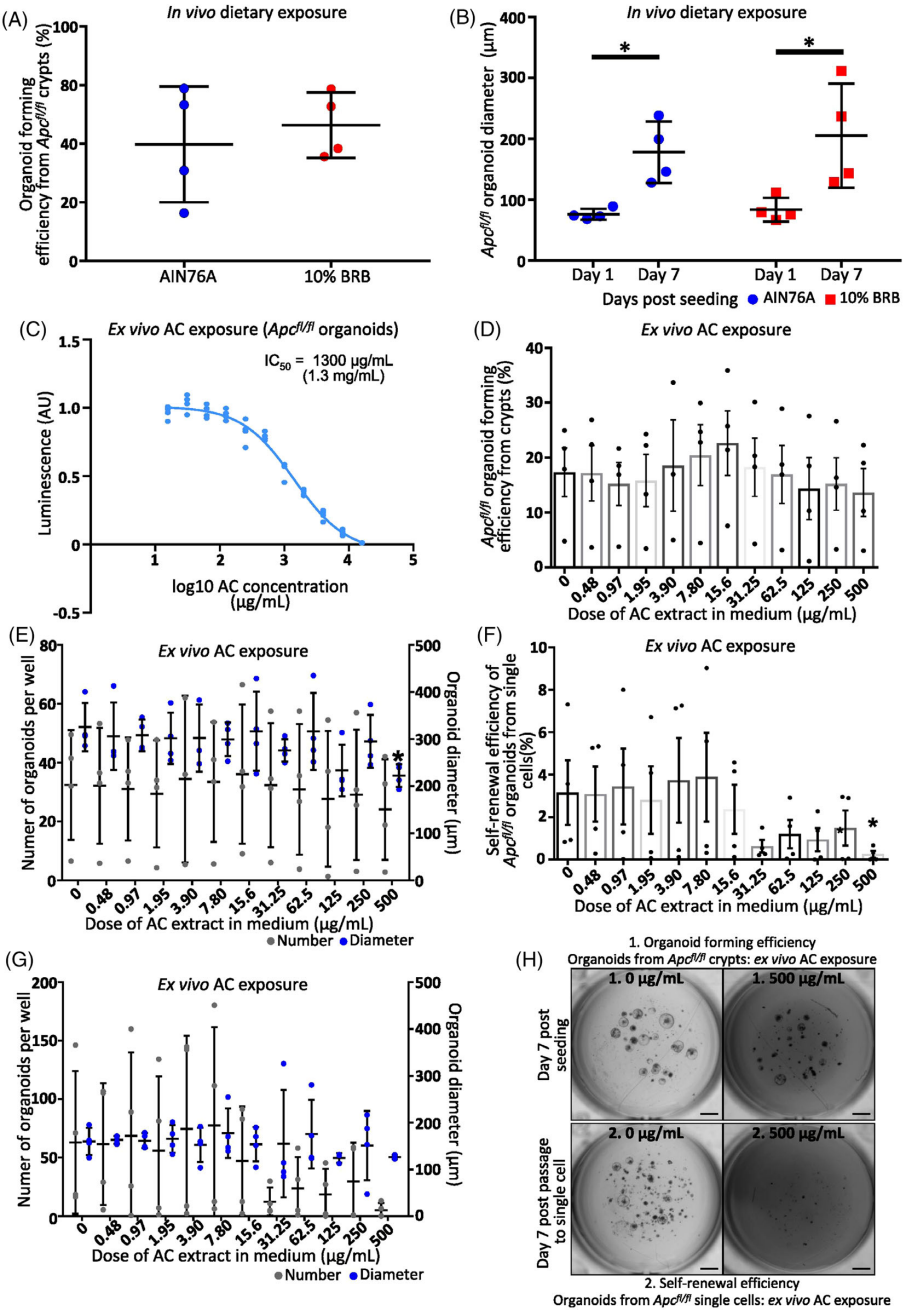
BRB‐derived anthocyanins (ACs) suppress *Apc* deficient ISCs cells. 2‐weeks in vivo BRB diet has no effect on A) organoid forming efficiency or growth B) of *Apc^fl/fl^* crypts (*N* = 4 mice lines). C) Viability curve demonstrating the sensitivity of ex vivo *Apc* deficient organoids to BRB‐derived ACs (IC_50_ = 1.3 mg mL^−1^, *N* = 4 technical replicates). D) ACs in the medium does not inhibit the ability of *Apc^fl/fl^* crypts to form ex vivo organoids (*N* = 3–4 mice lines). E) Organoids treated with 500 µg mL^−1^ AC medium are significantly smaller than control. F) Subsequent passage and 1 week exposure of organoids to ACs reduces the number of cells capable of forming a new organoid in a dose dependent fashion (*N* = 3–4 mice lines) but has no effect on their growth G). H) *VillinCreER^T2^Apc^fl/fl^* organoids treated with 0 and 500 µg mL^−1^ BRB‐derived ACs for 1‐week and subsequently passaged and grown for a further week in AC containing medium (1 mm).

To understand the relationship between BRB diet and reduced ISC gene expression in *Apc^fl/fl^* cells in vivo we performed a stem cell functionality assay using 3D ex vivo organoid culture^[^ ^11^ ^]^ (Figure S1F). As expected, a 2‐week exposure to BRBs in the diet prior to *Apc* deletion, did not impair the ability of *Apc^fl/fl^* crypts to form organoids or impede their growth compared to control (**Figure** **4**A,B), indicating that despite a reduction in ISCs (Figure 1I,J) each crypt contains a functional ISC. Thus, we next set out to evaluate whether exposure to BRBs ex vivo influenced the viability and self‐renewal capacity of *Apc^fl/fl^* organoids. We treated *Apc^fl/fl^* organoids with increasing concentrations of BRB‐derived ACs (spanning novel concentrations from 15.6 to 16 000 µg mL^−1^) that have been previously used on several human CRC cell lines.^[^ ^12^ ^]^ Cell viability demonstrated that *Apc^fl/fl^* organoids are sensitive to BRB‐derived ACs in a dose‐dependent fashion (Figure 4C; IC_50_ = 1.3 mg mL^−1^). One‐week exposure of *Apc^fl/fl^* crypts to sub lethal low toxicity AC concentrations between 0 and 500 µg mL^−1^ indicated no differences in organoid forming potential but at 500 µg mL^−1^ organoids were significantly smaller than control (Figure 4D,E,H); reflecting either a reduction in ISC numbers or reduced proliferative capacity. To examine this, AC treated organoids were split to single cells and reseeded for organoid culture. Following reseeding there was a reduction in the percentage of organoid forming cells in a dose dependent fashion, which was significant at 500 µg mL^−1^ (Figure 4F,H). There was no significant alteration to organoid growth following passage, but this could be attributed to the number of countable organoids treated with 500 µg mL^−1^ AC (Figure 4G). Together this data demonstrates that BRB‐derived ACs at sub‐lethal levels reduce ISC number and activity.

### 2.4 BRB Exposure Reduces ISC Gene Expression in the Human Bowel and the Tumorigenicity of CRC Organoid Cells

**Figure 5:**
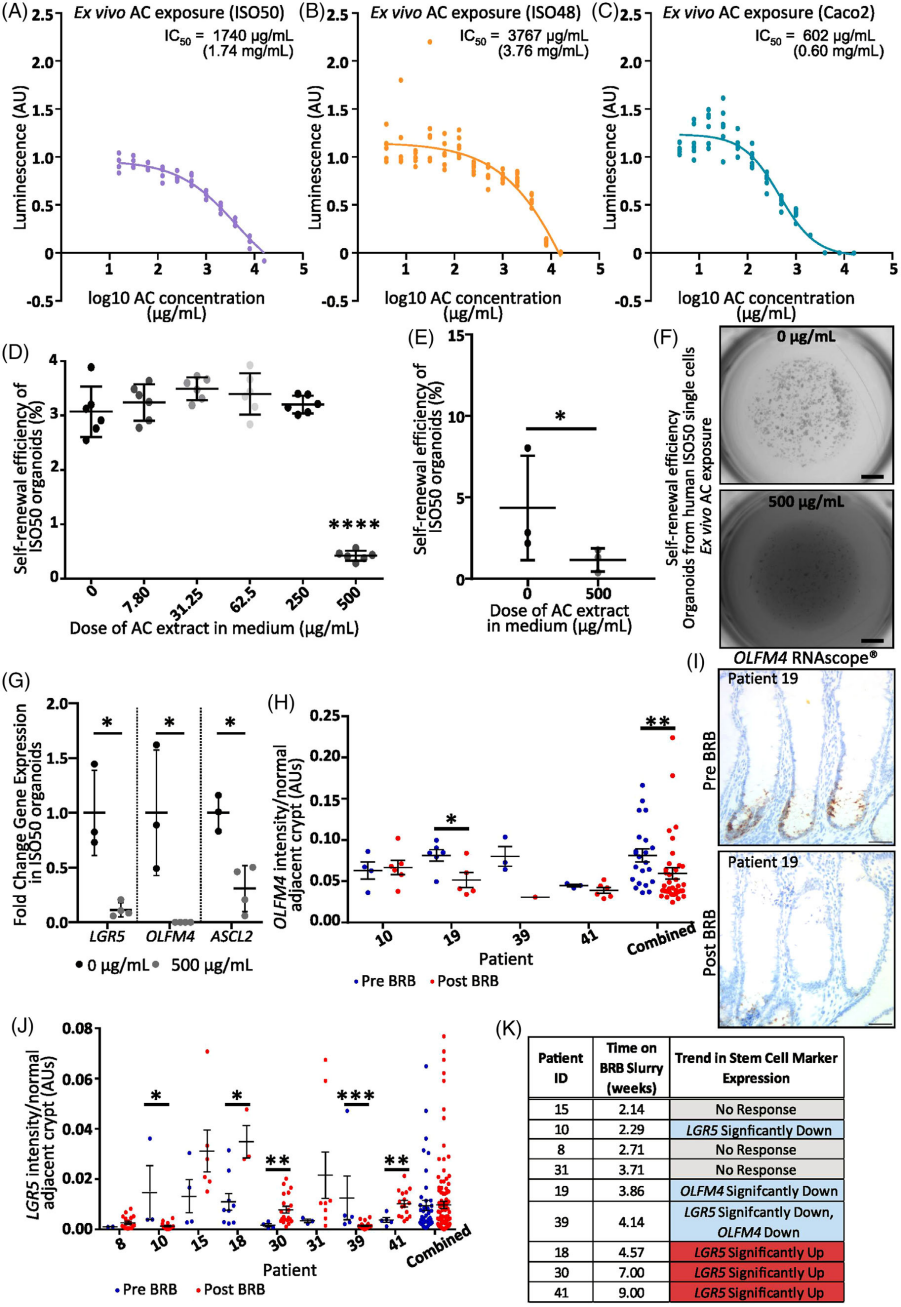
BRBs alter human stem cell marker gene expression and suppress human CRC cells in ex vivo culture. AC viability curves of ex vivo human CRC organoids A) ISO50 (IC_50_ = 1.74 mg mL^−1^); B) ISO48 (IC_50_ = 3.7 mg mL^−1^) and C) Caco2 cells (IC_50_ = 0.6 mg mL^−1^), ≥4 technical replicates each. 500 µg mL^−1^ AC exposure reduces the self‐renewal capacity of human ISO50 organoid cells (D; *N* = 6 technical replicates) (E; *N* = 3 different passages, note one biological replicate is the average of the data in panel D) (F; representative images of human ISO50 organoids exposed to 0 and 500 µg mL^−1^ BRB‐derived ACs for 1‐week after passage to single cell, 1 mm) and suppresses expression of ISC marker genes (G; *N* = 3–4 different passages). CRC patients administered oral BRBs (60 g day^−1^; 1–9 weeks) have a reduction in *OLFM4* expression (H; combined *N* = 8 patients/58 crypts and I; *OLFM4* brown, 50 µm) and differential *LGR5* expression (J; combined *N* = 10 patients/162 crypts) in CRC normal adjacent crypts. K) Table summarizing the length of time patients were on BRB intervention, showing that prolonged treatment results in changes to the ISC marker expression.

As a BRB diet has previously been shown to be effective in FAP^[^ ^7^ ^]^ and CRC patients^[^ ^6^ ^]^ we next investigated whether BRB treatment affects human ISCs. Using the ISO48, ISO50, and Caco2 cells grown in 3D we demonstrated that, akin to the mouse, they are sensitive to increasing AC concentrations in a dose‐dependent manner (**Figure** **5**A–C; ISO50 IC_50_ = 1.74 mg mL^−1^, ISO48 IC_50_ = 3.76 mg mL^−1^, and Caco2 IC_50_ = 0.6 mg mL^−1^). With the efficiency of ISO50 single cells to self‐renew significantly impaired following exposure to a sub‐lethal 500 µg mL^−1^ of ACs (Figure 5D), irrespective of passage number (Figure 5E,F). This reduction in ISO50 self‐renewal is supported by a significant reduction in gene expression of the ISC markers *LGR5*, *OLFM4*, and *ASCL2* in the organoids after a weeklong exposure to the AC extract (Figure 5G). To corroborate our data in humans we investigated whether intervention with oral BRBs, in CRC patients,^[^ ^6^ ^]^ altered ISC marker expression in normal CRC adjacent colonic tissues. Using RNAscope we stained for ISC markers *OLFM4* and *LGR5* before and after BRB intervention (Figure 5H–J). For *OLFM4*, 3/4 patients demonstrated a decrease in *OLFM4* staining intensity, significant in patient 19, and no alteration in patient 10 (Figure 5H). Due to the small tissue samples and number of countable crypts, analysis of *OLFM4* staining in crypts across all patients (*N* = 22 crypts/6 patients pre‐intervention, *N* = 36 crypts/8 patients post‐intervention) indicated a significant reduction in *OLFM4* expression post BRB intervention (Figure 5H,I). Changes in *LGR5* expression were inconsistent; it was unaltered in 3/8 patients, significantly increased in 3/8 patients and significantly decreased in 2/8 patients (Figure 5I), potentially due to the proximity of this tissue to the leading edge of a CRC affecting this Wnt target gene. Combined analysis indicated there was no meaningful change in *LGR5* expression across the cohorts (*N* = 45 crypts/10 patients pre‐intervention, *N* = 117 crypts/10 patients post‐intervention) (Figure 5J). As the original study was a biomarker assay, clinical endpoints were not collected. However, the stem cell marker changes were observed in patients who were exposed to BRB intervention for longer than 4 weeks in which significant improvement in prognostic biomarkers was most apparent (Figure 5K). The *OLFM4* data are consistent with our preclinical mouse data and despite low numbers indicate that BRBs impact on stem cell characteristics within the human bowel.

## 3 Conclusions

The nutritional polyphenols found in BRBs have been shown to have pleiotropic effects against CRC initiation and progression.^[^ ^5^, ^6^, ^7^, ^12^ ^]^ While there is strong evidence that BRBs reduce FAP polyp^[^ ^7^ ^]^ and tumor burdens,^[^ ^5^, ^6^ ^]^ alter the tumor microenvironment,^[^ ^13^, ^14^ ^]^ and reduces cancer‐related inflammation,^[^ ^5^ ^]^ there are limited studies on their effect on normal and malignant ISC populations. Given the known importance of an ISC as the cell of origin of CRC, prevention of CRC, in part, must relate to the number of ISCs required to maintain homeostasis. As ISCs play a role in how tissues adapt to alterations in dietary stimuli, there is increasing evidence that diet and obesity impacts on the ISC population and CRC risk. Thus, our data indicates that dietary exposure to BRBs prevents CRC by reducing the number of normal ISCs required for intestinal homeostasis and suppressing *Apc* deficient ISCs. It is important to note the impact on mutated cells, as while it is entirely plausible that decreasing ISCs can lead to decreased CRC risk the picture is not clear. ISCs follow a neutral drift model, in which each ISC has an equal probability of replacing its neighboring ISC or being replaced. *Apc* mutated ISCs follow a biased drift model, as it has an increase in clonal fitness which favors its retention. Reducing normal ISCs has the effect of increasing the fitness of a mutated ISC, thereby increasing the chance of it becoming fixed and forming a CRC.^[^ ^15^ ^]^ Therefore, for a reduction in CRC risk to be elicited by reducing normal ISC numbers, there must be an equivalent reduction in the fitness of any mutated ISC to prevent its increased likelihood of fixation, which we report here. It is of note then that the reduction in ISCs and ISC gene expression in BRB fed *AhCreApc^fl/fl^* mice is associated with increased *Ascl2* expression (Figure S1D, Supporting Information). *Ascl2* upregulation is associated with loss of *Lgr5+‐*ISCs^[^ ^9^ ^]^ and may represent emergence of *Ascl2^+^/Lgr5^−^* non‐ISC cells that migrate into the ISC compartment to dedifferentiate and replace the lost or damaged *Lgr5*+‐ISCs.^[^ ^9^ ^]^ As we demonstrated that the expansion of the ISC^[^ ^10^ ^]^ compartment, due to *Apc* loss, is attenuated by a BRB diet potentially the increased *Ascl2* expression may represent the generation of a stream of cells attempting to replace the functional stem cells which ultimately fails as they are continuingly being lost due to a diminished competitive advantage over normal neighbors. However, it remains to be seen whether the increase in *Ascl2* expression in the *AhCreApc^fl/fl^* would lead to rapid *Apc^−/−^* ISC expansion if BRBs were removed from the diet. In the healthy *AhCreApc^+/+^* model it is of note that *Ascl2* is not upregulated upon BRB exposure suggesting that the reduced numbers of ISCs is adequate to maintain cellular and tissue homeostasis and do not require replenishment from outside the ISC compartment.

The links between obesity and increased cancer risk are becoming well established.^[^ ^2^ ^]^ Importantly, we report that BRBs have no adverse effects on murine body weight over time consistent with other findings.^[^ ^16^ ^]^ Obesity is a major cause of chronic inflammation, a risk factor for metabolic syndromes and diabetes. There is some evidence in metabolic syndrome^[^ ^17^ ^]^ and pre‐diabetic patients^[^ ^18^ ^]^ that BRB interventions have positive effects on blood lipid levels and inflammation. Therefore, future studies should investigate the effects of BRBs on blood sugar levels, serum lipid profiles, and inflammatory markers in cancer. Furthermore, tumor‐induced weight loss is a common feature of cancer. We report weight loss as mice become symptomatic of disease, however mice treated with BRB diet tend to have on average a higher body weight than control suggesting that BRBs may maintain healthier weight even when symptomatic of disease which may contribute to our observed improved survival, however this needs further investigation.

In this study, we report that long‐term feeding of a BRB diet has no detrimental impact on overall health despite reductions in ISC number. This is in accordance with reports indicating that intestinal homeostasis is not perturbed following the complete ablation of Lgr5‐positive stem cells in mice,^[^ ^19^, ^20^ ^]^ while the significant increase in enteroendocrine cells in mice exposed to BRB is likely due to their roles as chemo‐sensors in the gut.^[^ ^21^, ^22^ ^]^ A previous study demonstrated that mice fed HFD had significantly lower numbers of enteroendocrine cells in the small intestine,^[^ ^4^ ^]^ thus it is possible that this cell population may increase in response to a change in dietary stimuli, in this instance, BRB diet. In addition, in the 2011 phase 1 clinical trial studying the effects of BRB slurry in CRC patients, it was reported that patients showed differential response to the BRB intervention, which was in part related to the length of time that patients were on the BRB treatment.^[^ ^6^ ^]^ This may account for our observed patient variation in colonic *LGR5* expression. The data also suggests that prolonged exposure (>4 weeks) to BRBs impacts the ISC population, which could correlate with the positive changes in epigenetic biomarkers previously reported.^[^ ^6^ ^]^ Additionally, it is important to note that the ISCs changes are observed in the normal adjacent tissue and thus may reflect *LGR5* driven repair, therefore it would be important to distinguish whether upregulation of *LGR5* expression reflects an increase in individual ISCs or an increase in Wnt activity in the in situ ISCs. Finally, questions remain over how the ability of BRBs to reduce ISCs competes with the ability of a high‐fat diet to increase them, but overall, this data provides evidence that a diet high in anthocyanin‐containing fruit may augment current CRC therapies and support campaigns to adopt a healthier lifestyle.

## 4 Experimental Section

Below was a summary of experiments, detailed information was available in supplementary material.

### Animal Experiments

Work was approved by a UK Home Office Project licence (30/3279) and reported in accordance with institutional and NC3R(UK) ARRIVE guidelines. Mixed sex outbred C57Bl6/J mice (11–19 weeks) were used with the following transgenes: *Apc^fl^*,^[^ ^23^ ^]^ *AhCre*,^[^ ^8^ ^]^ *VillinCreER^T2^*,^[^ ^24^ ^]^ and *Lgr5‐EGFP‐IRES‐CreER^T2^* (*Lgr5CreER^T2^*).^[^ ^25^ ^]^ Mice were randomly assigned to their respective diets from 2‐weeks prior to Cre activation until sacrifice at either a specific time point or a humane endpoint when symptomatic of disease (Figure S1A, Supporting Information).

### Dosage Information

Freeze‐dried BRB powder (10%) was pelleted into mouse AIN76A (Dyets Inc., USA; BRB diet) at the expense of sucrose.^[^ ^5^ ^]^ 10% rodent diet equated to ≈85 g of freeze‐dried BRB powder, equivalent to ≈0.9 kg of fresh BRBs. Mice were fed ad libitum their respective from 2‐week prior to *Apc* loss until the end of their experimental studied. For ex vivo organoid studies a BRB‐derived anthocyanin (AC) preparation containing cyanidin‐3‐O‐glucoside, cyanidin‐3‐O‐xylosylrutinoside, and cyanidin‐3‐O‐rutinoside^[^ ^12^ ^]^ was used. At 500 µg mL^−1^ the AC extract equaled ≈0.0147 g freeze‐dried BRB powder, or 0.084 g fresh berry.

### Cell Analysis

For immunohistochemistry (IHC), tissue was fixed in 10% neutral buffered formalin (Sigma, UK) and processed by conventional means. The following antibodies were used to stain for: *Apc* deficient cells anti‐β‐catenin (Transduction Lab #610154); apoptosis anti‐cleaved caspase 3 (CC3; CST #9661); for proliferation and migration anti‐BrdU (BD Biosciences #347580); proliferation anti‐Ki67 (Abcam #ab16667); Paneth cells anti‐lysozyme (ThermoScientific #RB372‐A1), and ISCs anti‐GFP (CST #2956S). Protocols were available upon request. Enteroendocrine and goblet cells were stained with grimelius^[^ ^26^ ^]^ and alcian blue,^[^ ^27^ ^]^ respectively. mRNA was visualized via in situ hybridization^[^ ^28^ ^]^ or RNAScope. Cellular analysis was performed within small intestinal tumors or on a total of 25 crypts from the first 5 cm of small intestine. Tumor burden was determined as a ratio of the area of nuclear β‐catenin positivity versus the total crypt area in longitudinal sections.

### Expression Analysis

RNA was isolated from 0.5 cm of frozen mouse small intestinal tissue (≈5 cm distal to stomach) or human organoids and transcribed using Superscript III (Invitrogen, UK). Relative gene expression analysis was carried out using SYBR green fast master mix (Applied Biosystems), primer sequences available on request, or TaqMan Universal PCR Master Mix with Assay on Demand probes (see Supplemental Information).

### In Vitro/Ex Vivo Analysis

For stemness and self‐renewal assays, the study used mouse organoids generated from intestinal crypts,^[^ ^11^ ^]^ human CRC organoid lines ISO48 and ISO50 (Cellesce, UK), and the Caco2 cell line. For in vivo stemness assays (Figure S1F, Supporting Information), *VillinCreER^T2^Apc^fl/fl^* mice were fed for 2‐weeks prior to induction. Mice were harvested 3 d.p.i and 200 crypts per well were plated in Matrigel in organoid culture medium. Medium was replaced every 2 days and organoid number determined at day 7 (Gelcount, Oxford Optronix, UK). For self‐renewal efficiency, organoids (mouse *N* = 3–4 lines; human *N* = 3 different passages) were cultured with AC medium for 7 days before dissociation into single cells using TrypLE (ThermoFisher Scientific, UK). Plates were seeded with 8000 cells per well in the presence of AC^[^ ^12^ ^]^ and ROCK inhibitor (Stem Cell Technologies, UK; first 3 days only, 10 µM final concentration). The number of organoids formed at day 7 was used to determine self‐renewal efficiency (Figure S1F, Supporting Information). For cell viability analysis, 8000 single cells were plated in normal medium and AC extract added at day 4. On day 7 post‐seeding, viability was assessed using CellTiter‐Glo (Promega, UK) and a CLARIOstar plate reader (BMG Labteck, UK).

### BRB Clinical Trial

The clinical trial was approved by the Institutional Review Boards of the Ohio State University Comprehensive Cancer Center and the University of Texas, San Antonio. All patients accrued to the trial had a diagnosis of CRC.^[^ ^6^ ^]^ Biopsies of CRC adjacent normal tissue (<2 mm) were obtained 24 h prior to administration of BRB. 20 g freeze‐dried berry powder was mixed with 100 mL of water and consumed orally three times a day (60 g day^−1^ total (equivalent to ≈7% rodent BRB diet) 6 h^‐1^ apart) for 1–9 weeks. Patients remained on BRBs until 12–36 h prior to surgery to remove tumor. At surgery, an additional three adjacent normal tissue biopsies were taken from each patient. All tissue specimens were fixed in formalin and assessed by a medical pathologist.

### Statistics

Genotype and/or diet controls were used throughout this study and no bias was applied during husbandry, tissue sampling, or outcome analysis. On graphs if not indicated otherwise, *p* values are: ∗*p* < 0.05; ∗∗*p* < 0.01; ∗∗∗*p* <0.001, with data points indicated on graph with mean ± standard deviation (SD).

## Author Contributions

Study concept and design by S.M., K.R.G., and LP. Data acquisition and/or material support by S.M., E.T., M.K., A.T.H., A.V.D., P.P., L.S.W., and C.N. Data analysis by S.M., T.W., and L.P. Manuscript drafted by S.M. Critical revision of manuscript by S.M., L.S.W., O.J.S., and L.P. Funding acquisition by K.R.G. and L.P.

## Conflict of Interest

The authors declare no conflict of interest.

## Supporting information



Sup Figure 1(A). A schematic timeline illustrating feeding and induction regimes for the animal models and time points for tissue analysis (βNF ‐ β‐naphthoflavone; TAM ‐ Tamoxifen; IP ‐ intraperitoneal injection). 10% freeze‐dried BRB diet has no significant effect on weight gain in (B) *Lgr5CreER^T2^Apc^+/+^* or (C) *Lgr5CreER^T2^Apc^fl/fl^* mice over time when compared to control fed mice; AIN76A *Apc^+/+^* N = 6 mice per timepoint; *Apc^+/+^* BRB N = 6 mice per timepoint except at day 80 where N = 3 mice; *Apc^fl/fl^* AIN76A: N = 11, 11, 8, 6 and 7 mice at days ‐14, 0, 16, 30 and at death respectively; *Apc^fl/fl^* BRB: N = 10, 10, 10, 8 and 10 mice at days ‐14, 0, 16, 30 and at death respectively. (D) BRB diet suppresses *Olfm4* ISC gene expression but increases *Ascl2* gene expression in induced *AhCreApc^fl/fl^* mice (N = 3‐4 mice, mean±SEM). (E) Representative In situ images demonstrating a reduction in *Olfm4* expression in the *AhCreApc^fl/fl^* intestinal crypts following BRB exposure (100 µm). (F) Graphical representation of the *Apc^fl/fl^* organoid forming and self‐renewal assay methodology utilised in this study in the presence of BRB diet or BRB‐derived anthocyanin extract. ∗P<0.05; ∗∗P<0.01; ∗∗∗P<0.001.

Supporting Information.

## Data Availability

The data that support the findings of this study are available from the corresponding author upon reasonable request.
