# Supplementary figures and images for: Modification of Diet to Reduce the Stemness and Tumorigenicity of Murine and Human Intestinal Cells
Source: Mol Nutr Food Res. 2022 Aug 31;66(19):2200234. doi: 10.1002/mnfr.202200234 (PMC9539894; doi:10.1002/mnfr.202200234)

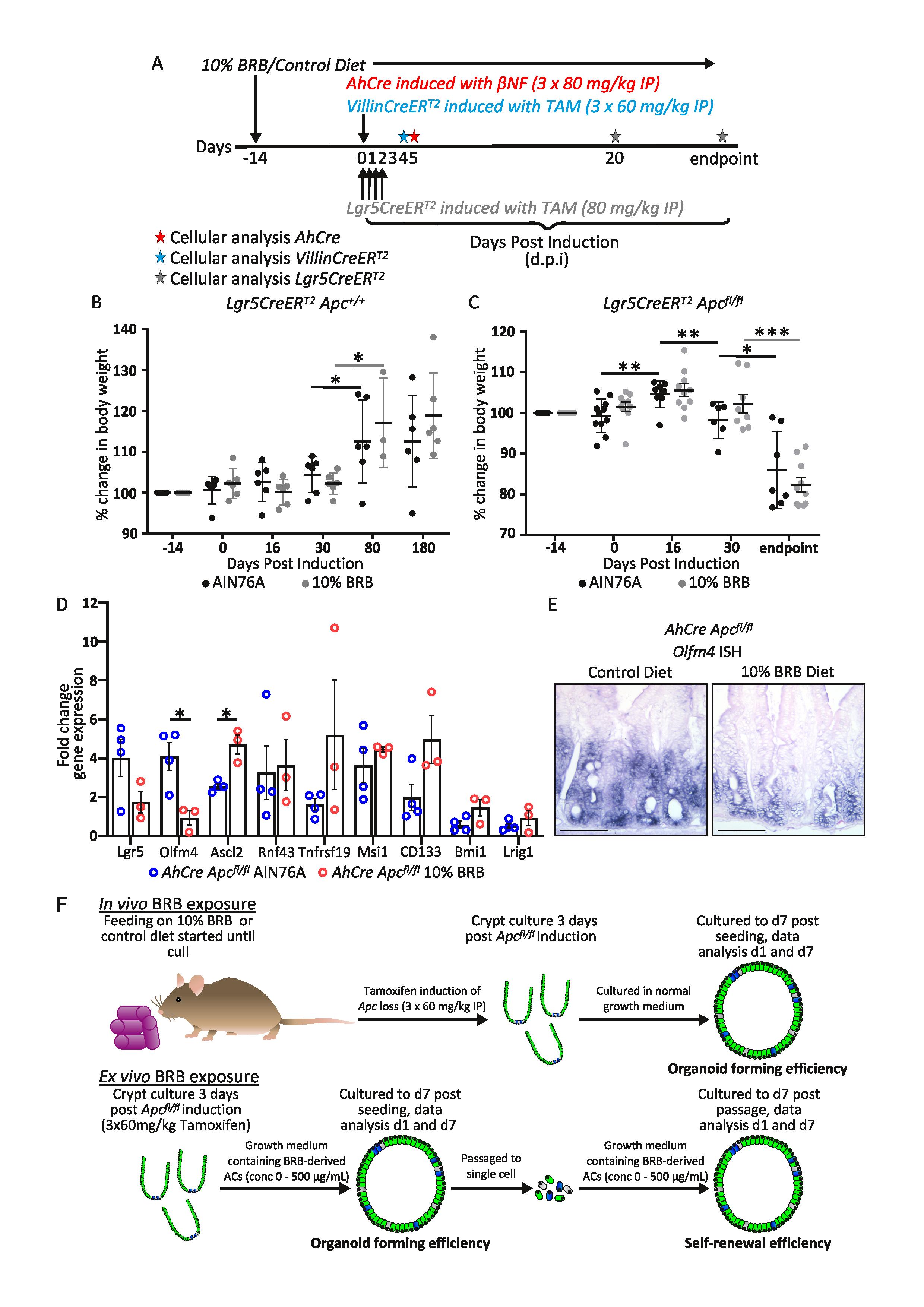

Supplement: Supplementary file 1 — Sup Figure 1(A). A schematic timeline illustrating feeding and induction regimes for the animal models and time points for tissue analysis (βNF ‐ β‐naphthoflavone; TAM ‐ Tamoxifen; IP ‐ intraperitoneal injection). 10% freeze‐dried BRB diet has no significant effect on weight gain in (B) Lgr5CreERT2Apc+/+ or (C) Lgr5CreERT2Apcfl/fl mice over time when compared to control fed mice; AIN76A Apc+/+ N = 6 mice per timepoint; Apc+/+ BRB N = 6 mice per timepoint except at day 80 where N = 3 mice; Apcfl/fl AIN76A: N = 11, 11, 8, 6 and 7 mice at days ‐14, 0, 16, 30 and at death respectively; Apcfl/fl BRB: N = 10, 10, 10, 8 and 10 mice at days ‐14, 0, 16, 30 and at death respectively. (D) BRB diet suppresses Olfm4 ISC gene expression but increases Ascl2 gene expression in induced AhCreApcfl/fl mice (N = 3‐4 mice, mean±SEM). (E) Representative In situ images demonstrating a reduction in Olfm4 expression in the AhCreApcfl/fl intestinal crypts following BRB exposure (100 µm). (F) Graphical representation of the Apcfl/fl organoid forming and self‐renewal assay methodology utilised in this study in the presence of BRB diet or BRB‐derived anthocyanin extract. ∗P<0.05; ∗∗P<0.01; ∗∗∗P<0.001. [file MNFR-66-0-s001.jpg]
