# Supplementary material for: Modification of Diet to Reduce the Stemness and Tumorigenicity of Murine and Human Intestinal Cells
Source: Mol Nutr Food Res. 2022 Aug 31;66(19):2200234. doi: 10.1002/mnfr.202200234 (PMC9539894; doi:10.1002/mnfr.202200234)
Supplement: Supplementary file 2 — Supporting Information. [file MNFR-66-0-s002.pdf]

Supplemental Experimental Section

Animal experiments

Animals were maintained on an outbred C57Bl6/J background and housed in a standard facility under a 12hr light cycle, with *ad libitum* water and RM3(E) diet (expanded diet, Special Diet Services UK). Genotyping conditions are available upon request. *AhCre* and *VillinCreER*^{T2} mice were induced by 3 intraperitoneal (IP) injections within 12hrs of 80 mg/kg β-naphthoflavone or 60 mg/kg tamoxifen dissolved in corn oil (Sigma, UK), respectively. *Lgr5CreER*^{T2} was induced by IP injection of 80 mg/kg tamoxifen once daily for 4 days. Cohort sizes were based on power calculations performed using the G*Power software[(1)] considering variability observed in previous experiments. For the short-term comparisons cohorts of N=3-4 provided the power to detect an effect size of 3.2-4.2, for survival cohorts of N=15 we had achieved a power of 0.71. For survival experiments disease symptoms were recognized as weight loss, pale feet, bloating, or piloerection. Blinding of the researchers to the animal cohorts were not possible due to the differences in diet colours (normal chow: beige, AIN76A: cream/ off-white and BRB diet: purple), however, researchers were blinded to animal ID, genotype and diet allocation for subsequent downstream analyses.

Diet Information

AhCre mice (19 weeks) received either RM3(E) control or BRB diet, *VillinCreER*^{T2} and *Lgr5CreER*^{T2} mice (11-15 weeks) were fed either AIN76A control or BRB diet.

Tissue staining and immunohistochemistry

Histological analysis was performed on images taken using an Olympus BX43 light microscope or scanned images from a Zeiss Axio Scan.Z1 slide scanner. Data were acquired using either automated HALO 2.0 software (Indica Labs) or performed manually from microscope images or in Aperio ImageScope (v12.4.0.5043). For IHC and RNAscope® crypt regions of interest were manually selected using Fiji ImageJ and the mean staining intensity per whole crypt was obtained using an in-house script; previously tested for accuracy by correlation with manual scoring, providing intensity values in a range between 0 and 1 arbitrary units. For representative images, processing adjustments were performed equally.

Expression Analysis

Mouse RNA was extracted using Trizol (Life Technologies) and DNAse treated (TURBO DNA-*free*™ kit, Ambion, UK). Human RNA was extracted using Trizol (Life Technologies) with an additional glycogen co-precipitant (250 µg/mL, Invitrogen) step to maximise yield and DNase treated (RQ1 RNase-Free DNase Promega). RNA integrity and concentration were confirmed using a Thermo Scientific NanoDrop™ 2000. Analysis was performed on the QuantoStudio7 Flex real-time PCR (Applied Biosystems, UK). Assay on demand probes used were *Lgr5* Mm00438890_m1, *Olfm4* Mm01320260_m1, *ActB* 4352933E, *LGR5* Hs00969422_m1, *OLFM4* Hs00197437_m1, *ASCL2* Hs00270888_s1 and *GAPDH* Hs02786624_g1) Data were normalised against housekeeping gene expression. All samples were run in triplicate.

In vitro/ex vivo analysis

Cell viability was determined with CellTitre-Glo® using murine *Apc* deficient organoids (1 biological replicate with 4 technical replicates performed in the same experiment), human-derived Wnt driven CRC organoids (ISO50 (1 biological replicate with 4 technical replicates performed in the same experiment) and ISO48 (1 biological replicate with 6 technical replicates performed in the same experiment), and the Caco2 cell line (1 biological replicate with 6 technical replicates performed in the same experiment)). For each cell line the luminescence reads were normalised to the average of the 0µg/mL replicates then plotted in GraphPad software and analysed using Non-linear fit regression model (log(inhibitor) vs. response -- Variable slope (four parameters)). 10 to the power of the interpolated x value mean was calculated to get the IC_{50}.

Statistics

Genotype and/or diet controls were used throughout this study and no bias was applied during husbandry, during tissue sampling or during outcome analysis. Data were analysed using GraphPad software, v7.02 (La Jolla, CA). Data were tested for normality using a Shapiro-Wilk test and then compared using 1 (N=<4) or 2-tailed (N≥4) unpaired t tests for normally distributed data or non-parametric tests (Mann-Whitney or Kolmogorov-Smirnov to determine significant differences in cell distribution) for data that did not follow a normal distribution. Survival data were analysed using the Log-Rank (Mantel-Cox) test. On graphs if not indicated otherwise, P values are: ∗P < 0.05; ∗∗P < 0.01; ∗∗∗P <0 .001, with

data indicated on graph with mean ± standard deviation (SD). Figures were constructed using Scribus v1.4.7 (GNU general public license). Any deviations are indicted in the figure legends.

References

1. F. Faul, E. Erdfelder, A.-G. Lang, A. Buchner, *Behavior Research Methods* **2007**, *39*, 175.
